# Intraductal papillary mucinous neoplasm of the intrahepatic bile duct: a review of literature and a rare case report

**DOI:** 10.3389/fsurg.2023.1133375

**Published:** 2023-05-25

**Authors:** Lehat Zibari, Muhammad S. Naseer, Het Patel, Hosein Shokouh-Amiri, Gregory Wellman, David Dies, Veron Browne, Gazi B. Zibari

**Affiliations:** Department of Surgery and Transplant, Willis-Knighton Health System, Shreveport, LA, United States

**Keywords:** IPMN—intraductal papillary mucinous neoplasm, biliary diseases, intrahepatic bile duct, case report, MRCP—magnetic resonance cholangiopancreatography, CBD—common bile duct, carcinoma

## Abstract

**Background:**

Intraductal papillary neoplasm of the bile duct is a rare variant of bile duct tumors, which is characterized by papillary or villous growth inside the bile duct. Having papillary and mucinous features such as those found in pancreatic intraductal papillary mucinous neoplasm (IPMN) is extremely rare. We report a rare case of intraductal papillary mucinous neoplasm of the intrahepatic bile duct.

**Case report:**

A 65-year-old male Caucasian with multiple comorbidities presented to the emergency room with moderate constant pain at the right upper quadrant (RUQ) abdomen for the last several hours. On physical examination, he was found to have normal vital signs, with icteric sclera and pain on deep palpation at the RUQ region. His laboratory results were significant for jaundice, elevated liver function tests and creatinine, hyperglycemia, and leukocytosis. Multiple imaging studies revealed a 5 cm heterogeneous mass in the left hepatic lobe that demonstrated areas of internal enhancement, mild gall bladder wall edema, dilated gall bladder with mild sludge, and 9 mm common bile duct (CBD) dilatation without evidence of choledocholithiasis. He underwent a CT-guided biopsy of this mass, which revealed intrahepatic papillary mucinous neoplasm. This case was discussed at the hepatobiliary multidisciplinary conference, and the patient underwent an uneventful robotic left partial liver resection, cholecystectomy, and lymphadenectomy.

**Conclusion:**

IPMN of the biliary tract may represent a carcinogenesis pathway different from that of CBD carcinoma arising from flat dysplasia. Complete surgical resection should be performed whenever possible because of its significant risk of harboring invasive carcinoma.

## Introduction

A variety of mucin-secreting, papillary, and cystic lesions of the intra- and extrahepatic biliary tract have been reported with increased frequency (1, 2). Intraductal papillary neoplasm of the bile duct (IPNB) is a rare variant of bile duct tumors, which is characterized by papillary or villous growth inside the bile duct (3). There are pathological similarities between these lesions and pancreatic intraductal papillary mucinous neoplasm (IPMN) (4, 5). We report a rare case of intraductal papillary mucinous neoplasm of the intrahepatic bile duct.

## Case description

**Figure 1:**
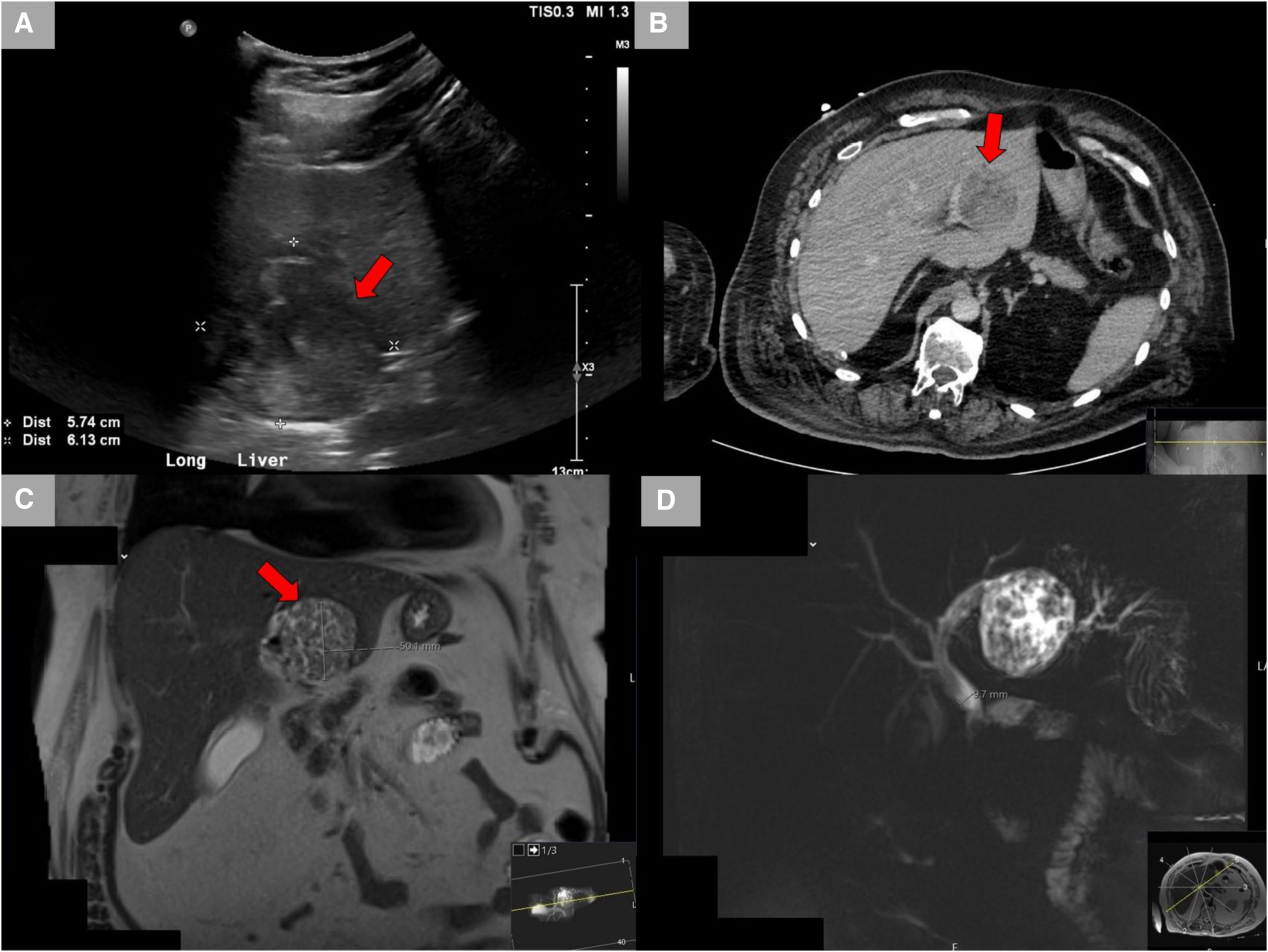
Radiological studies. (**A**) Ultrasound showing the left hepatic lobe heterogeneous area. (**B**) CT scan with contrast showing a lesion in the left lobe of the liver demonstrates areas of internal enhancement and a mildly distended gall bladder. (**C**) MRCP showing a lesion in the left hepatic lobe measuring up to 5 cm. (**D**) MRCP showing mild gall bladder wall edema without additional signs of cholecystitis and mild CBD dilatation of 9 mm without evidence of choledocholithiasis. CT, computed tomography; MRCP, magnetic resonance cholangiopancreatography; CBD, common bile duct.

**Figure 2:**
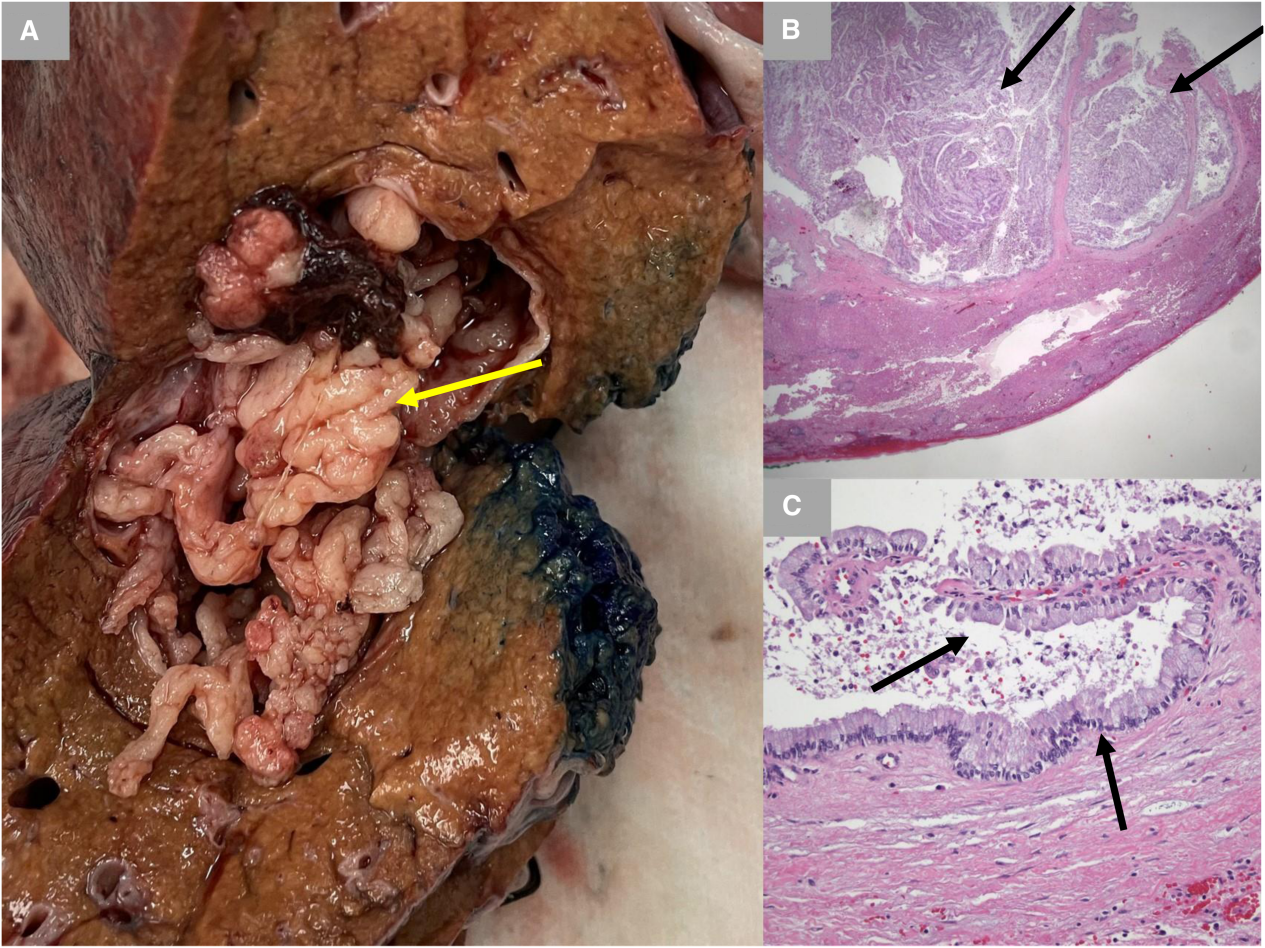
Gross pathology and histology. (**A**) Gross pathology with inked margins. IPMN is shown with a yellow arrow. (**B**) Low-power photomicrograph demonstrating papillary fronds emanating from the cyst lining (black arrows) (hematoxylin and eosin stain; 12.5× original magnification). (**C**) High-power photomicrograph demonstrating a very low-grade papillary mucinous epithelium (black arrow) histologically identical to that typically seen with pancreatic intraductal papillary mucinous neoplasm. There is no evidence of underlying ovarian-type stroma (hematoxylin and eosin stain; 200× original magnification). IPMN, intraductal papillary mucinous neoplasm.

**Figure 3:**
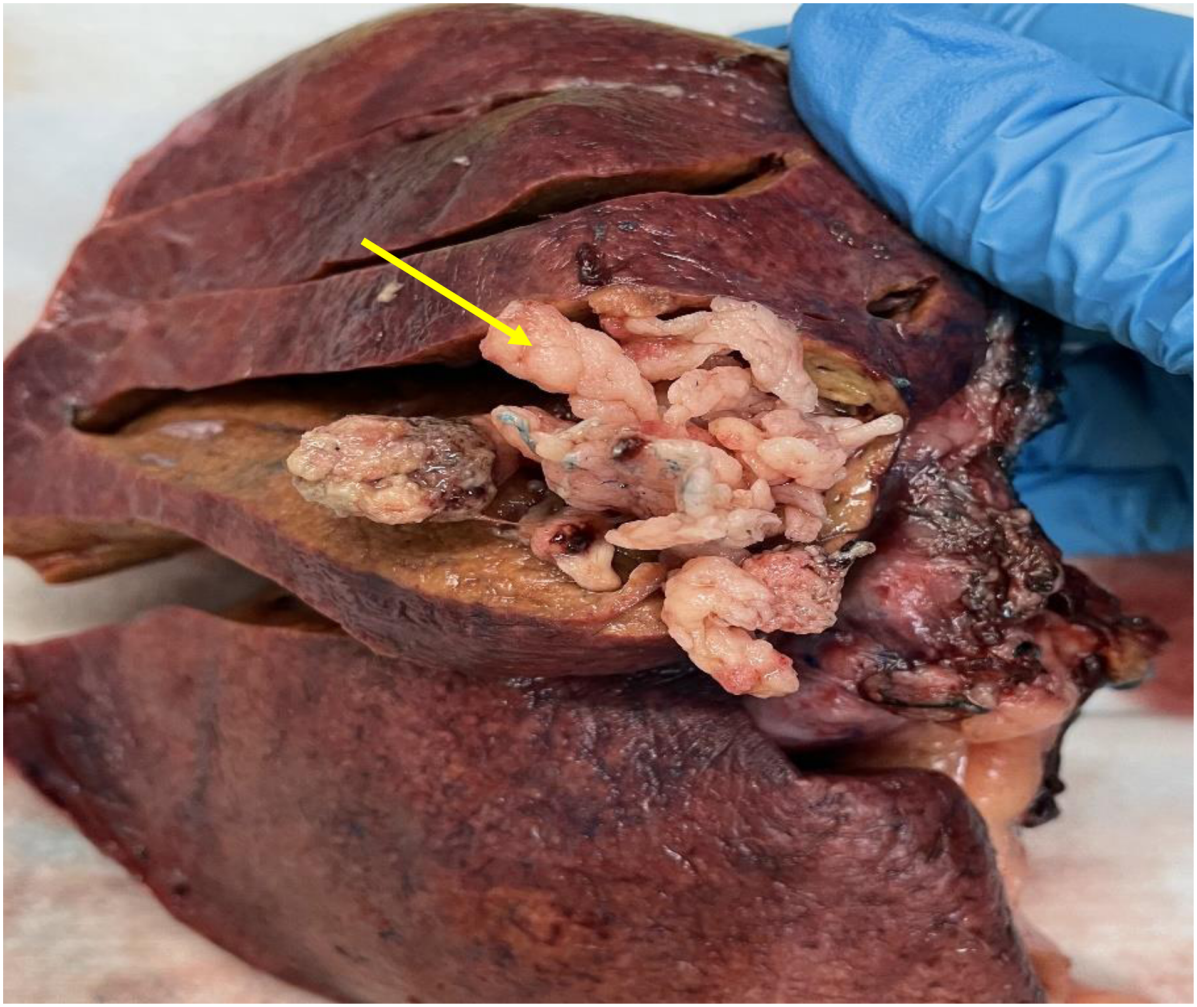
Gross pathology image.

A 65-year-old male Caucasian with a history of multiple comorbid conditions presented to the emergency room with complaints of moderate constant pain at the right upper quadrant (RUQ) abdomen for the last several hours. Differential diagnoses of symptomatic cholelithiasis, cholangitis, and liver abscess were entertained. On physical examination, he was found to have normal vital signs, with icteric sclera and pain on deep palpation at the RUQ region. Upper abdominal ultrasonography (U/S) (Figure 1A) revealed a 6 cm heterogeneous mass in the left hepatic lobe and dilated gall bladder with mild sludge or stones. His laboratory results were significant for elevated liver enzymes, bilirubin and creatinine, hyperglycemia, and leukocytosis (Table 1). Subsequently, he had a hepatobiliary (HPB) iminodiacetic acid scan, which showed no excretion of hepatobiliary radionuclide related to either severe liver dysfunction or high-grade biliary obstruction. In addition, a computed tomography (CT) scan of the upper abdomen and pelvis (Figure 1B) was performed, which confirmed the U/S findings of the lesion in the left lobe of the liver and demonstrated areas of internal enhancement, and magnetic resonance cholangiopancreatography (MRCP) (Figures 1C,D) revealed mild gall bladder wall edema and a 9 mm common bile duct (CBD) dilatation of without evidence of choledocholithiasis, and it was consistent with an undetermined 5 cm mass in the left lobe of the liver. To confirm the pathology of the lesion, the referring physician performed a CT-guided biopsy, which revealed an intrahepatic papillary mucinous neoplasm. This case was discussed at the HPB multidisciplinary conference, and the patient was recommended to undergo left liver mass resection, cholecystectomy, and repair of incisional hernia. His comorbid conditions include cardiac disease, chronic obstructive pulmonary disease, diabetes mellitus, hypertension, and cerebrovascular accident. Cardiology evaluated the patient and declared him as a moderate surgical risk. He uneventfully underwent robotic left partial liver resection, cholecystectomy, lymphadenectomy, and repair of ventral hernia, and his hospital course was unremarkable. Gross pathology (Figure 2A) and histology (Figures 2B,C) revealed negative margins, and the 4.5 cm  ×  3.5 cm  ×  3.0 cm cystic structure from hepatic tissue represents the intrahepatic biliary IPMN (yellow arrow) (Figure 3) with low-grade dysplasia. The central aspect of the mass is comprised of pink-tan papillary tissue. There is no gross evidence of invasion (Figure 3). The remainder of the liver parenchyma is red-brown and firm. The patient came for regular postoperative follow-up and is still alive. The lesion had low-grade dysplasia and no malignant focus; however, the patient still underwent abdominal ultrasound after 3 months and a CT scan after 1 year. Also, he was followed up by his primary care doctor. His last visit to a hospital was on 20 March 2023 for non-IPMN or non–liver-related issues. He had a history of cerebro vascular accident (CVA) with dense right hemiparesis. His postoperative course was uneventful, other than a mild urinary tract infection for which he was prescribed antibiotics. He progressed well with physical therapy and was discharged to an inpatient rehab after 8 days of hospital stay. The patient had an extended stay at the hospital due to a lack of rehab beds. He was readmitted to the hospital from rehab due to a pulseless electrical activity (PEA) cardiac arrest for a short period, from which he successfully recovered and was discharged home with an outpatient rehab plan.

**Table 1:** Laboratory results.

	At admission (16/08/2021)	Preoperative (27/08/2021)	Postoperative (28/08/2021)	Before discharge (05/09/2021)
Total bilirubin (0.2–1.3), mg/dL	3.8	0.9	1.1	1.0
ALT (0–50), U/L	164	28	813	173
AST (3–45), U/L	275	27	950	91
ALP (38–126), U/L	298	109	148	142
Glucose (70–109), mg/dL	328	115	224	115
Creatinine (0.66–1.25), mg/dL	1.96	1.68	1.57	1.74
Albumin (3.5–5.0), g/dL	4.7	2.8	3.5	3.5
WBC count (3.1–9.7), 10^3^/µL	15.7	8.2	12.7	9.8

ALT, alanine aminotransferase; AST, aspartate aminotransferase; ALP, alkaline phosphatase; WBC, white blood cells.

## Discussion

In 2010, the World Health Organization had adopted and recognized IPNB as a distinct clinical and pathological classification (6). The highest incidence rate is reported in Southeast Asia, and this is likely due to the high incidence rate and endemic nature of hepatolithiasis and clonorchiasis in that region of the world (7, 8). It occurs in patients between the ages of 50 and 70 years. These patients usually present with RUQ abdominal pain, jaundice, and cholangitis (3, 7–9). A tumor may occur anywhere in the biliary tract including the intrahepatic bile duct, perihilar, distal common bile duct, and even cystic duct (10). The most common radiologic findings for IPNB are bile duct dilatation and intraductal masses, which can be recognized by U/S, CT, and magnetic resonance imaging (MRI) (3, 11, 12). In addition, direct cholangiography, such as endoscopic retrograde cholangiopancreatography (ERCP), percutaneous transhepatic cholangiogram (PTC), and cholangioscopy, is useful for the detection of mucobilia and can confirm the location and extent of tumor involvement (11–13).

Intrahepatic IPNBs are usually larger than pancreatic IPMNs, and approximately 40% of them show mucin hypersecretion, which tends to accumulate around papillary lesions inside bile ducts leading to dilatation and/or infection (14). IPNB is usually stratified according to specific pathological findings. Bennett et al. have mentioned in Oxford publications that IPNB can be divided into gastric, oncocytic, pancreatobiliary, and intestinal types based on their microscopic morphology (15). However, experts in Japan and Korea have developed a classification system with two subtypes based on different characteristics and disease prognosis (16), i.e., (1) intrahepatic and (2) extrahepatic. The intrahepatic type is similar to pancreatic IPMN, while the extrahepatic type has a more complex histological architecture and is associated with invasive cancer and a worse prognosis (17). Early detection and intervention are enhanced due to bile duct obstruction in the early stage of the disease (18). Hence, all patients with IPNB should be considered for surgical resection because papillary tumors and associated mucin often lead to recurrent attacks of cholangitis and obstructive jaundice, even if these lesions are benign. IPNB in patients without evidence of metastasis should be resected similar to intra- and extrahepatic cholangiocarcinoma. In addition, regional lymphadenectomy should be performed, and an intraoperative frozen section should be obtained to ensure the bile duct margins are tumor-free (19, 20).

IPNB are rarer variants of bile duct cancer, and they account for roughly 10% of all resectable cases (21). These lesions can occur anywhere in the biliary tree. Due to the exophytic nature and intraductal growth pattern of the lesions, patients with papillary cholangiocarcinoma appear to have better prognoses (22). Also, they share clinical and histologic features with pancreatic IPMN and may represent a carcinogenesis pathway different from that of CBD carcinoma arising from flat dysplasia. Complete surgical resection should be performed whenever possible because of the significant risk of harboring invasive carcinoma (7, 20).

## Conclusion

Intraductal papillary neoplasm of the biliary tract carries the risk of obstructing the biliary tract and increases the risk of progressing to invasive carcinoma. All patients should undergo early complete surgical resection in order to improve the prognosis.

## Data Availability

The raw data supporting the conclusions of this article will be made available by the authors, without undue reservation.
